# Molecular Epidemiology and Characterization of Carbapenem-Resistant *Klebsiella pneumoniae* Isolated from Urine at a Teaching Hospital in Taiwan

**DOI:** 10.3390/microorganisms9020271

**Published:** 2021-01-28

**Authors:** Yuarn-Jang Lee, Chih-Hung Huang, Noor Andryan Ilsan, I-Hui Lee, Tzu-Wen Huang

**Affiliations:** 1 Division of Infectious Diseases, Department of Internal Medicine, Taipei Medical University Hospital, Taipei 11031, Taiwan; yuarn438@yahoo.com.tw; 2 Division of Infectious Diseases, Department of Internal Medicine, School of Medicine, College of Medicine, Taipei Medical University, Taipei 11031, Taiwan; 3 Graduate Institute of Biochemical and Biomedical Engineering, National Taipei University of Technology, Taipei 10608, Taiwan; chhuang@mail.ntut.edu.tw; 4 International Master/Ph.D. Program in Medicine, College of Medicine, Taipei Medical University, Taipei 11031, Taiwan; noorandryanilsan@gmail.com; 5 Department of Microbiology and Immunology, School of Medicine, College of Medicine, Taipei Medical University, Taipei 11031, Taiwan; sos810712@gmail.com; 6 Graduate Institute of Medical Sciences, College of Medicine, Taipei Medical University, Taipei 11031, Taiwan

**Keywords:** antimicrobial resistance, carbapenem-resistant mechanism, extended-spectrum β-lactamase, carbapenemase, urinary tract infection

## Abstract

Urinary tract infections (UTIs) are common in clinics and hospitals and are associated with a high economic burden. Enterobacterium *Klebsiella pneumoniae* is a prevalent agent causing UTIs. A high prevalence of carbapenem-resistant *K. pneumoniae* (CRKP) has emerged recently and is continuing to increase. Seventeen urinary CRKP isolates collected at a teaching hospital in Taiwan from December 2016 to September 2017 were analyzed to elucidate their drug resistance mechanisms. Two-thirds of the isolates were obtained from outpatients. Antimicrobial susceptibility tests demonstrated multidrug resistance in all the isolates. Multilocus sequence typing analysis showed high diversity among the isolates. PCR analysis demonstrated the presence of carbapenemases in three isolates. All isolates carried at least one other extended-spectrum β-lactamase, including TEM, DHA, and CTX-M. Fifteen isolates contained mutations in one of the outer membrane porins that were assessed. The expression levels of the *acrB* and/or *oqxB* efflux pump genes, as determined by qRT-PCR, were upregulated in 11 isolates. Six isolates might have utilized other efflux pumps or antimicrobial resistance mechanisms. These analyses demonstrated a highly diverse population and the presence of complex resistance mechanisms in urinary isolates of *K. pneumoniae*.

## 1. Introduction

Urinary tract infections (UTIs) are common bacterial infections in individuals of all ages and are associated with a high economic burden [1]. UTIs are clinically classified as uncomplicated and complicated UTIs. Uncomplicated UTIs usually affect healthy individuals without any abnormality of urinary tract structures. Complicated UTIs are commonly associated with host defenses and the existence of drainage devices or catheters [2]. Although UTIs are usually not life-threatening and can be treated by antibiotics, the high frequency of UTIs and the tendency for UTI recurrence place heavy economic burdens in healthcare systems [3]. Bacterial pathogens are the major causative agents of UTIs. Uropathogenic *Escherichia coli* (UPEC) is the most common bacterial pathogen, accounting for 75% of uncomplicated UTIs and 65% of complicated UTIs. *Klebsiella pneumoniae* is the second most common causative agent in uncomplicated UTIs and ranks third in complicated UTIs [3].

*K. pneumoniae* is an opportunistic pathogen that commonly causes a wide spectrum of infections, including pneumonia, bacteremia, urinary tract infection, and liver abscess [4]. Classical *K. pneumoniae* strains are multidrug-resistant and rarely cause infections other than UTIs in healthy individuals. Nonclassical hypervirulent *K. pneumoniae* strains are susceptible to most antibiotics but may cause invasive infections in both healthy and immunocompromised people in the community [5]. Recently, new *K. pneumoniae* strains possessing both multidrug resistance and hypervirulence were reported in China [6], elevating concerns regarding *K. pneumoniae* infections.

*K. pneumoniae* strains are commonly observed in urine specimens in addition to *E. coli*. *K. pneumoniae* accounts for approximately 5% of either nosocomial- or community-acquired UTIs [7], in which the highest level of antimicrobial resistance was found [8,9]. Therefore, *K. pneumoniae* was classified as a critical pathogen by the WHO in 2017 [10]. Two primary mechanisms conferring carbapenem resistance in *K. pneumoniae* have been identified. The first mechanism is the presence of extended-spectrum β-lactamases (ESBLs) combined with the loss of outer membrane proteins OmpK35 and OmpK36 or the overexpression of efflux pumps [11]. Several types of ESBLs, including TEM, SHV, CTX-M, or AmpC (DHA or CMY), have been reported in *K. pneumoniae* [12]. The other primary resistance mechanism is the presence of carbapenemases. Five carbapenemases, namely, KPC, NDM, IMP, VIM, and OXA-48, have been identified in *K. pneumoniae* [13].

Nationwide surveillance of the prevalence of carbapenem-nonsusceptible *K. pneumoniae* (CnSKP) in the past two decades in Taiwan has been published [14,15,16]. The trend of ESBL- or AmpC-producing *K. pneumoniae* increased in Taiwan from 2002 to 2012 [15]. Two major ESBLs, CTX-M and DHA, were detected in the same isolates of CnSKP with high frequencies (85.6%) from 2010 to 2012 [14]. The dominant carbapenemase was KPC, accounting for 77% of 457 carbapenemase-producing *K. pneumoniae* from 2012 to 2015 [16]. However, these studies did not focus on CnSKP from urine. In addition, research on the molecular features and resistance mechanisms focused mainly on drug-resistance enzymes.

To elucidate the molecular epidemiology of carbapenem-resistant *K. pneumoniae* (CRKP) from urine specimens in Taiwan and to investigate their resistance mechanisms, we collected 137 CRKP isolates from a teaching hospital in Taipei city from December 2016 to September 2017. We analyzed 17 isolates from the urine. Antimicrobial susceptibility tests showed that these isolates were primarily multidrug-resistant and were only susceptible to colistin and tigecycline tested. Common β-lactamase genes and emerging carbapenemase genes were detected by PCR. Only 3 of the isolates produced the emerging carbapenemases including KPC and VIM. Mutation analysis showed that most isolates (13/17, 76%) contained amino acid substitutions or frameshift mutations in two outer membrane proteins OmpK35 and OmpK36. Moreover, known efflux pumps of some isolates were overexpressed, and membrane permeability was reduced in some isolates. This study exhibits the complexity of the carbapenem resistance mechanisms of CRKP obtained from urine specimens in Taiwan.

## 2. Materials and Methods

### 2.1. Clinical Isolates of K. Pneumoniae

CRKP isolates from any kind of specimen have been routinely collected at Taipei Medical University Hospital (TMUH), a teaching hospital with 800 beds in Northern Taiwan, since December 2016. Bacterial isolation and identification were performed by automated biochemical tests in the clinical laboratory at TMUH. The criteria for carbapenem-resistant isolates were nonsusceptibility to any of the three tested carbapenems including imipenem, ertapenem, and meropenem. A total of 137 CRKP isolates were collected until September 2017. Among 50 isolates from urine specimens, 17 isolates were selected based on nonsusceptibility to meropenem (≥2 μg/mL) and/or to imipenem (≥2 μg/mL) and cefmetazole (≥32 μg/mL), and their resistance mechanisms were investigated in this study.

### 2.2. Antimicrobial Susceptibility Test

Antimicrobial susceptibility tests (ASTs) were performed by using microbroth dilution methods, disc diffusion methods, or Etest. Routine ASTs in the Laboratory of Clinical Microbiology at TMUH were performed by using the BD Phoenix™ Automated Identification and Susceptibility Testing System (BD Diagnostics System, Sparks, MD, USA) to obtain an antibiogram of each isolate. Several classes of antibiotics for the application to *K. pneumoniae* include β-lactams, β-lactams combined with their inhibitors, aminoglycosides, fluoroquinolones, and folate pathway inhibitors. Confirmation tests of the selected antibiotics were performed by disc diffusion methods. The minimal inhibitory concentration (MIC) of colistin was determined by using a manual microbroth dilution assay ranging from 0.25 to 128 μg/mL. For tigecycline, the MIC was measured by using ETEST^®^ (bioMérieux, Marcy-l’Étoile, France) on Muller-Hinton agar. All ASTs, except tigecycline, were interpreted according to the criteria of Enterobacteriaceae in the Clinical and Laboratory Standard Institute (CLSI) guideline (2018) [17]. The interpretation of tigecycline followed the European Committee on Antimicrobial Susceptibility Testing (EUCAST) (2018).

### 2.3. Genomic DNA Extraction

The DNA template was prepared by using a Wizard^®^ Genomic DNA Purification Kit (Promega, Madison, WI, USA). Bacterial cells were inoculated into 3 mL tryptic soy broth and cultured in an orbital shaker at 37 °C. After overnight incubation, the bacterial cells were harvested by centrifugation at 15,000 g for 2 min. Genomic DNA purification followed the manufacturer’s instructions. In brief, bacteria were lysed by the addition of Nucleic Lysis Solution and the mixture was incubated at 80 °C for 5 min. After RNase treatment at 37 °C for 30 min, the protein was precipitated from the cell extract by adding Protein Precipitation Solution and placing the sample on ice for 5 min after vortexing. The protein was removed after centrifugation at 17,000 g for 5 min. The supernatant containing the DNA was transferred into a new Eppendorf tube for ethanol precipitation by the addition of isopropanol. After centrifugation, the DNA pellet was washed with 70% ethanol to remove the residual salt. The remaining 70% ethanol of the DNA pellet was completely aspirated and evaporated. The genomic DNA was resuspended in 100 μL of DNA Rehydration Solution. The quality and quantity of genomic DNA were measured by using a NanoDrop^®^ ND-1000 spectrophotometer. The integrity of genomic DNA was evaluated by agarose gel electrophoresis.

### 2.4. Multilocus Sequence Typing and Relatedness Analysis

Sequence types of clinical isolates were determined based on the Pasteur scheme [18], which analyzes the allelic profiles based on seven conserved genes (*gapA*, *infB*, *mdh*, *pgi*, *phoE*, *rpoB*, and *tonB*). Capsule type was determined by using the *wzi* allele [19]. The sequences of the primer pairs applied on these amplicons are listed in Table S1. Each amplicon was amplified by PCR and subjected to Sanger sequencing. The PCR reaction mixtures were composed of 5 μL of 2x Taq PCR mix (Taigen Bioscience Corporation, Taipei, Taiwan), 100 nM of each primer, and 10 ng of genomic DNA from each isolate. The amplification program was initiated at 95 °C for 5 min; 32 cycles of 95 °C for 30 s, 50 °C or 60 °C for 30 s, and 72 °C for 45 s; and a final extension at 72 °C for 7 min. The sequence results were inspected by using Chromas Lite 2.1 (Technelysium Pty Ltd., Brisbane, Queensland Australia). Only qualified regions of amplicons were chosen for the determination of gene alleles and the assignment of each sequence type (ST) based on the allelic profile in the *Klebsiella* MLST database (https://bigsdb.pasteur.fr/klebsiella/klebsiella.html, the last access date 2020/04/15). The diagram of the relatedness of clinical CRKP isolates from urine at TMUH was generated by using the goeBURST algorithm in PHYLOViZ Online [20].

### 2.5. Detection of Extended-Spectrum β-Lactamase Genes and Carbapenemases

Extended-spectrum β-lactamase (ESBL) genes and carbapenemase genes were screened by polymerase chain reaction (PCR) amplification. The six ESBL genes included *bla*_TEM_, *bla*_SHV_, and group 1, group 2, group 9, and group 8/25 of *bla*_CTX-M_. Five carbapenemase genes, *bla*_IMP_, *bla*_KPC_, *bla*_NDM_, *bla*_OXA-48_, and *bla*_VIM_, were used. The primer pairs of each targeted gene are listed in Table S2. The PCR mixture contained 5 μL of 2x Taq PCR mix (Taigen Bioscience Corporation, Taipei, Taiwan), 200 nM of each primer, and 5 ng of genomic DNA as the template. The amplification program was as follows: initial denaturation at 95 °C for 5 min; 32 cycles of 95 °C for 30 s, 60 °C for 30 s and 72 °C for 1 min; and a final extension at 72 °C for 5 min. For carbapenemase genes, the annealing temperature was optimized to 55 °C. The PCR products were analyzed after agarose gel electrophoresis and staining with nucleic acid dyes. The amplicon size was determined by comparison to the DNA marker.

### 2.6. Sequence Analysis of the Outer Membrane Proteins OmpK35 and OmpK36

The mutations of two outer membrane proteins, OmpK35 and OmpK36, were analyzed by a combination of PCR amplification and Sanger sequencing. The primer pairs for these two genes are shown in Table S3. PCR amplification was performed by using 2x Taq PCR mix (Taigen Bioscience Corporation, Taipei, Taiwan) with its optimal buffer condition, 100 nM of each primer, 100 μM deoxynucleotides, and 10 ng genomic DNA from each isolate as the template. The PCR program was the same as that for the β-lactamase genes except that the annealing temperature was optimized to 60 °C and the extension time was 1 min. Each amplicon was subjected to Sanger sequencing by using both forward and reverse primers. High-quality DNA sequences were selected for comparison with references by using BLASTX. The accession number of the OmpK35 reference was AFR33751.1 in NCBI. Four types (from A to D) of *ompK36* genes have been described [21]. The NCBI accession numbers representing types A to D of OmpK36 are QIR56615.1, QHW96390.1, QIV29771.1, and QEI51044.1, respectively.

### 2.7. Measurement of Gene Expression

Five milliliters of each bacterial culture grown in tryptic soy broth was harvested at the exponential phase (OD_600_ 0.5–0.7). After centrifugation, the cell pellet was resuspended in 0.5 mL RNA*later* solution (Invitrogen, Thermo Fisher, USA) and incubated for 1 h at room temperature. The total RNA of treated bacterial cells was extracted by a PureLink™ RNA Mini Kit (Thermo Fischer Scientific, Waltham, MA, USA). The procedures were performed according to the manufacturer’s instructions. A total of 10 μg RNA was treated with DNase I (Lucigen Corporation, Middleton, WI, USA) to remove contaminated genomic DNA. The treated RNA was purified by using RNA Clean and Concentrator™-5 (Zymo Research, Irvine, CA, USA) and stored at −80 °C until use. The concentration and quality of purified RNA were determined by using a Nanodrop ND-1000. The integrity of the purified RNA was analyzed by agarose gel electrophoresis. The complement DNA was synthesized by using KAPA RT Mix (Kapa Biosystems, Wilmington, MA USA) according to the manufacturer’s instructions. Quantification of 3 target genes (*acrB*, *oqxB*, and *ramA*) and 1 reference gene (*rpoB*) was performed by using the SensiFAST™ SYBR Hi-ROX Kit (Meridian Life Science, Memphis, TN, USA) in an Applied Biosystems 7300 Real-Time PCR System (Thermo Fischer Scientific, Waltham, MA, USA) with the following cycling condition: 95 °C for 3 min; 40 cycles of 95 °C for 3 s, 55 °C for 20 s and 72 °C for 27 s; and a final extension at 72 °C for 5 min. The primer pairs for the quantification of these genes are listed in Table S3. The Ct (threshold cycle) of each amplicon was determined based on the automatic setting. The relative gene expression level of each gene was calculated by using the 2^−∆∆Ct^ method [22] for comparison with the reference strain (*K. pneumoniae* BCRC 13883) and the housekeeping gene, *rpoB*. Biological triplicates were performed for the measurement of relative gene expression levels. Both the graphs and the statistical analyses were produced by GraphPad Prism 5.

### 2.8. Fluorescent Dye Accumulation Assay

Envelope permeability was evaluated by the Hoechst (H) 33342 dye accumulation assay described in Jimenez-Castellanos et al. [23]. Briefly, a bacterial culture was inoculated in 4 mL of LB broth and incubated in a 37 °C shaker until reaching the log phase (OD_600_ 0.5–0.7). The bacterial cells were harvested by centrifugation at 6000 g for 10 min. The cell pellets were resuspended and adjusted to an OD_600_ equal to 0.5 with PBS buffer. The fluorescent dye H33342 was added to the bacterial suspension at a final concentration of 2.5 μM. A 180-μL aliquot of each isolate was placed into each well of black flat-bottom plates (Costar, Corning, NY, USA). Fluorescence was measured every 5 min for 70 min by using a Varioskan Flash microplate reader (Thermo Fischer Scientific, Waltham, MA, USA) with excitation and emission filters at 355 nm and 460 nm, respectively. Two efflux pump inhibitors, phenylalanine-arginine β-naphthylamide (PAβN) and cyanide-chlorophenylhydrazone (CCCP), were separately added into the previously described bacterial suspension with the H33342 dye at final concentrations of 25 μg/mL and 25 µM, respectively. Fluorescence was measured as previously described. The relative fold changes of dye accumulation were calculated by comparing the fluorescence intensities in the presence of inhibitors to those in the absence of inhibitors for each isolate.

## 3. Results

### 3.1. Carbapenem-Resistant K. pneumoniae from Urine

A survey of carbapenem-resistant *K. pneumoniae* (CRKP) was initiated at Taipei Medical University Hospital (TMUH), a regional teaching hospital in northern Taiwan, in December 2016. After 10 months, a total of 137 CRKP isolates were collected, and 40% of the isolates originated from urine specimens. Seventeen CRKP isolates from urine were determined to be nonsusceptible to either meropenem or to both imipenem and cefmetazole. Characterization and antimicrobial susceptibility tests of these isolates are shown in Table 1. Two isolates (T1060405 and T1060431) were isolated from the same patient over a 9-day interval, whereas the remaining 15 isolates were isolated from individual patients. The ages of the patients ranged from 47 to 95 years, and 70% of them were elderly patients (≥65 years old). Over 60% of the individuals (10/16) were outpatients. All 17 isolates were collected in August and September 2017 and were classified as multidrug-resistant based on antimicrobial susceptibility tests (Table 1). Only a few antibiotics, including cefepime, meropenem, amikacin, levofloxacin, colistin, and tigecycline, were more than 50% effective against these bacteria, and the susceptibility of the bacteria to the remaining tested antibiotics was reduced. In particular, the most susceptible antibiotic was colistin (88.2%), followed by amikacin (82.4%) and tigecycline (76.5%). Two isolates (T1060405 and T1060431) from the same patient showed resistance to both colistin and tigecycline.

### 3.2. Capsule Types and Population Structure of CRKP from Urine

**Figure 1:**
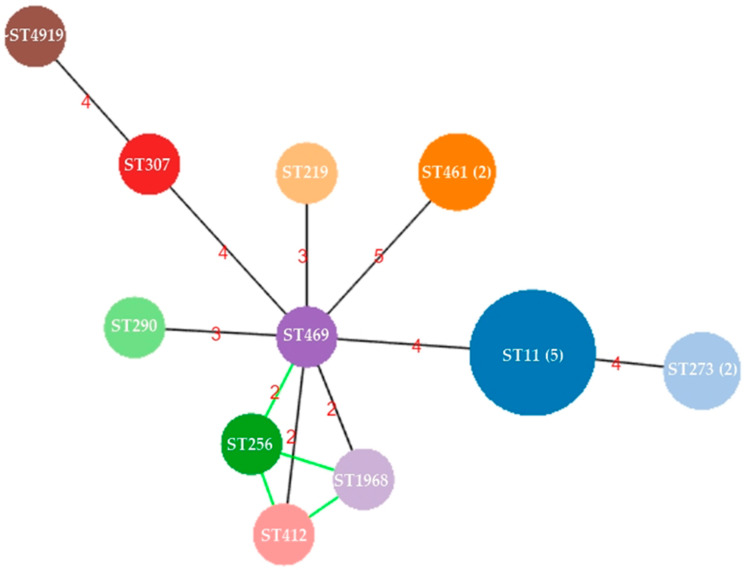
The relatedness among 17 carbapenem-resistant *K. pneumoniae* isolates from urine at Taipei Medical University Hospital. The sequence types of these isolates were determined based on the Pasteur scheme of multilocus sequence typing. A diagram of relatedness among the 17 carbapenem-resistant *K. pneumoniae* (CRKP) isolates was illustrated by using PHYLOViZ Online. The size of each circle indicates the number of isolates in each ST. More than one isolate in each ST is shown in parentheses. The numbers within the lines indicate the difference in allele numbers between two STs.

A total of 10 ST types were identified among the 17 CRKP isolates from urine (Table 2). The dominant ST type was ST11, accounting for 29.4% (5/17). The remaining 12 isolates were classified into 9 different STs. Ten capsule types were identified among the 17 isolates. The capsule type corresponded to a specific ST, except for two capsule types (KL47 and KL64), which were found in the ST11 isolates. The capsule type of ST256 was KL47, the same type as one of the ST11 isolates. One isolate (T1060498) was close to ST4919 or ST5154 due to the difference in the *tonB* allele, and its capsule type was undetermined because no PCR product of the *wzi* gene was amplified. The relationship among these 17 isolates was constructed based on MLST results and shown in Figure 1. The founder ST of these CRKP isolates was ST469. Four ST types (ST256, ST412, ST469, and ~ST1968/ST5154) exhibited variants of two alleles, *phoE* and *tonB*, and were identified in 4 individual patients at different times. All these isolates showed diverse ST types, with at least 2 locus variants indicating different sources of these isolates.

### 3.3. Detection of Acquired β-Lactamases and Mutation Analysis of OmpK35 and OmpK36

Five acquired carbapenemase genes and genes encoding extended-spectrum β-lactamases (ESBLs) were screened by PCR. Moreover, the alteration of two outer membrane porins, OmpK35 and OmpK36, was investigated through PCR and Sanger sequencing. Nucleotide sequences encoding the two-outer membrane porins OmpK35 and OmpK36 were obtained and analyzed. Only three isolates carried either KPC- or VIM-type carbapenemases, and no NDM-, IMP-, or OXA-48-type carbapenemases were observed among the 17 CRKP isolates. The KPC-positive isolate (T1060125) belonged to ST11 with capsule type KL47, which is a common ST associated with high resistance. The other two isolates (T1060144 and T1060393) carrying the VIM carbapenemase both belonged to ST461-KL51 and showed high resistance to all tested cephalosporins and carbapenems except intermediate resistance to cefmetazole. All 3 isolates also contained both SHV- and CTX-M-type group 9, but only the KPC-carrying isolate contained a frameshift mutation of the *ompK35* gene. This result indicated that multiple resistance mechanisms cooccurred in this KPC-positive isolate.

All 17 isolates had SHV-type β-lactamases, and 70.6% (12/17) of the isolates carried TEM-type β-lactamases. Over 80% of the isolates (14/17) harbored DHA β-lactamases. Half of the isolates (52.9%, 9/17) contained CTX-M-type β-lactamases, of which group 9 CTX-M β-lactamases were dominant. No group 2 or group 8/25 CTX-M-type β-lactamases were identified among the 17 isolates. Frameshift mutations of OmpK35 were identified in one-third of the isolates (35.3%, 6/17), including all 5 ST11 isolates. In contrast to OmpK35, types A to D of OmpK36 have been described [23]. Approximately half of the type A or type D OmpK36 porins were classified as wild type, indicating that they possessed the same sequences as the corresponding references. The loss of function of OmpK36 was mainly found in type C across the different ST types. In total, 13 isolates contained ESBLs and lacked one of these two porins. Taken together, these data indicate that the carbapenem resistance mechanism was primarily mediated through the existence of ESBLs combined with the loss of the outer membrane porins OmpK35 and OmpK36 in the urine isolates. However, two isolates without porin changes or carbapenemases might have developed carbapenem resistance mediated by another mechanism.

### 3.4. Role of Efflux Pumps in Carbapenem Resistance

#### 3.4.1. Expression of Efflux Pumps and the Related Regulator RamA

**Figure 2:**
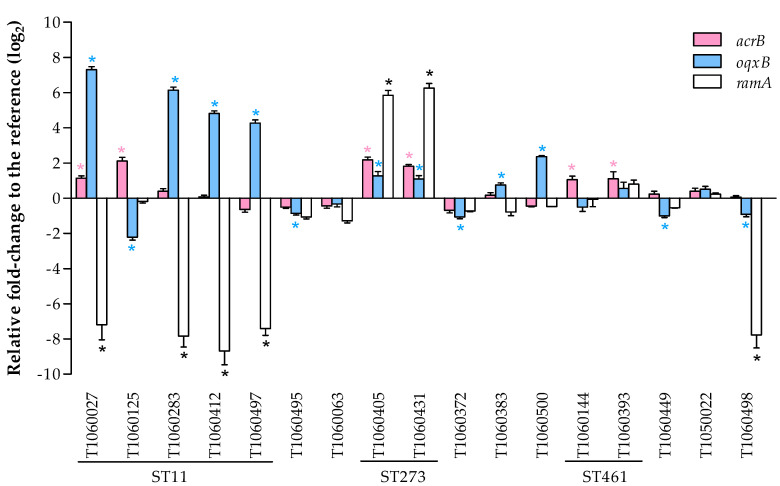
The relative gene expression of 2 efflux pump genes, *acrB* and *oqxB*, and a transcriptional activator, *ramA,* was measured by using quantitative reverse transcriptase PCR and compared with the expression of a housekeeping gene, *rpoB*. The bar height represents the mean relative fold-change relative to the reference strain, *K. pneumoniae* BCRC 13883, in triplicate. The error bar represents the standard error with a 95% confidence interval compared to the reference strain. The asterisk (*) indicates a significant difference with *p* ≤ 0.05.

The overexpression of efflux pumps has been shown to be another mechanism conferring carbapenem resistance in *K. pneumoniae*. The gene expression levels of the main efflux pumps (*acrB* and *oqxB*) and a key regulator (*ramA*) were investigated in our clinical isolates (shown in Figure 2). Four ST11-KL64 isolates showed significant downregulation of *ramA* and overexpression of *oqxB*, which is known to confer antibiotic resistance [24]. In contrast, the KPC-positive ST11-KL47 isolate showed upregulation of *acrB* and downregulation of *oqxB*. Only 2 ST273 isolates (T1060405 and T1060431 from the same patient) with wild-type OmpK35 and OmpK36 porins showed overexpression of the *ramA* gene and efflux pumps, which conferred colistin and tigecycline resistance (Table 1). Another isolate (T1060498), which was close to a recently reported ST4919 and contained integration of the insertion sequence at the 5’ end of the *ompK36* gene, exhibited downregulation of the *ramA* and *oqxB* genes. Two VIM (+) ST461 isolates showed slightly increased *acrB* expression. The remaining 7 isolates showed diverse expression patterns of the *oqxB* gene. Although all 7 isolates contained ESBLs, only 4 of the isolates carried defective mutations resulting in the loss of function of porins. Interestingly, the remaining 3 isolates with ESBLs had wild-type OmpK35 and wild-type or missense mutation of OmpK36, possibly indicating new mechanisms for carbapenem resistance.

#### 3.4.2. Membrane Permeability

**Figure 3:**
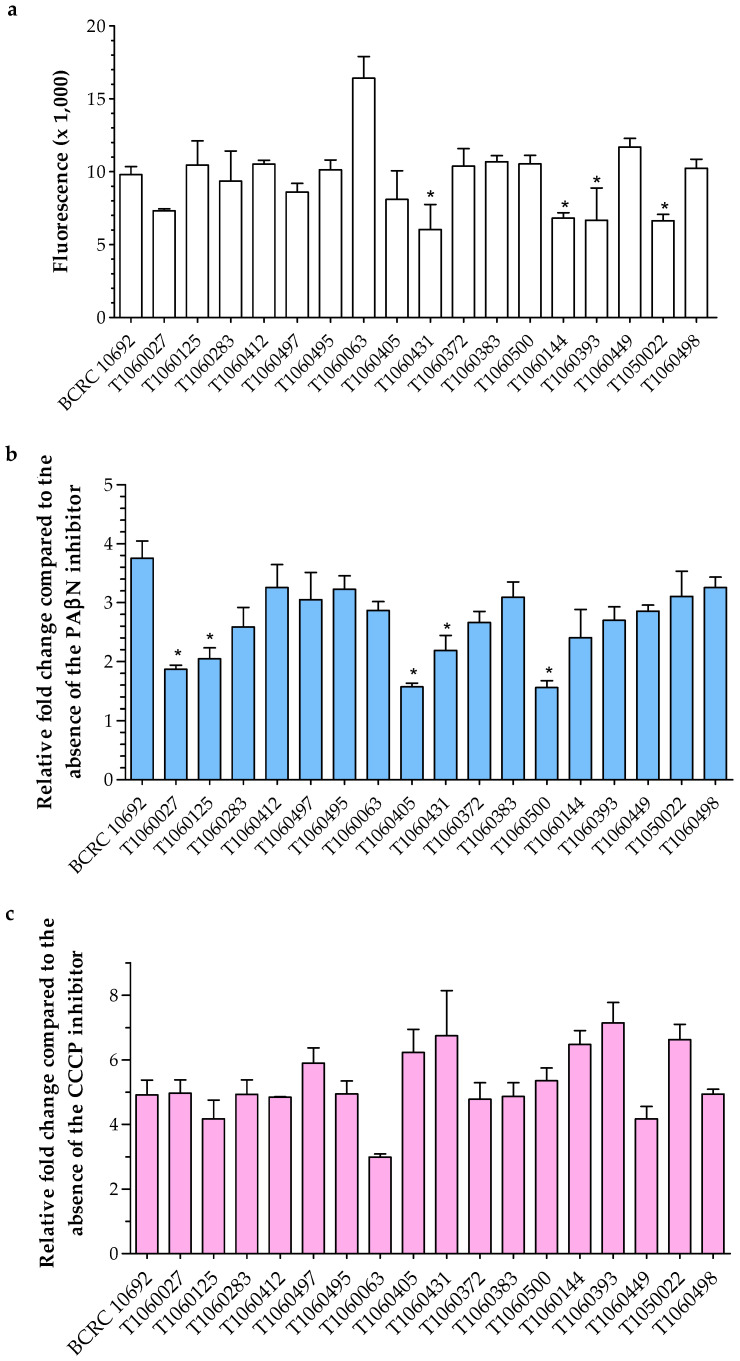
Membrane permeability by using fluorescence dye (Hoechst 33342) accumulation in the absence and presence of the efflux pump inhibitors (EIs) PAβN and CCCP. (**a**) Fluorescence values of each isolate were detected in the absence of EIs. (**b**) and (**c**) Fold changes in each isolate were calculated from the fluorescence values in the presence of individual EIs divided into those in the absence of EIs. The asterisk (*) indicates a significant difference compared to the reference BCRC 10692 (*p* ≤ 0.05).

The membrane permeability of each isolate was measured by detecting fluorescent dye accumulation in bacterial cells. Only one isolate (T1060063) had lower membrane permeability than the reference strain, and most isolates showed fluorescence intensity similar to that of the reference (Figure 3a). In particular, 6 isolates with low fluorescence intensities indicated that their membrane permeability was higher than that of the reference (Figure 3a). In the presence of efflux pump inhibitors, either PAβN or CCCP, the membrane permeability of bacterial isolates was blocked, resulting in the accumulation of fluorescent dye inside bacterial cells. In general, all clinical isolates showed low fold-changes in the presence of the inhibitor PAβN (Figure 3b). This result suggested that other RND-type efflux pumps might be more active in the clinical isolates than in the reference. Meanwhile, most isolates had similar fold changes to the reference in the presence of CCCP (Figure 3c). The effect of CCCP was less pronounced in the isolate T1060063, which had low permeability. A slight increase in fold-change was mainly found in isolates with high permeability (comparison with Figure 3a,c).

## 4. Discussion

In this study, we investigated the molecular epidemiology and antibiotic resistance mechanisms of CRKP isolates recently obtained from urine specimens at a teaching hospital in Taipei, Taiwan. Seventeen isolates, mostly from elderly outpatients, exhibited multidrug resistance. Multilocus sequence typing showed no dominant sequence types or clonal groups, implying that these isolates have diverse origins. The most abundant type (5/17) was ST11, a Clonal Complex 258, which was present in 60% of carbapenemase-producing *K. pneumoniae* isolates in China [25]. Four ST11 isolates (ST11-KL64) harbored the same type of ESBL β-lactamases, the same mutation in the OmpK35 gene, and exhibited similar expression profiles of *oqxB* and *ramA*. This finding implies that the isolates are closely related. ST11-KL64 also dominated in the CRKP isolates previously collected from 2010 to 2013 in Taiwan [26]. This clade also had enhanced virulence, which emerged after 2016 in China [27]. The relatedness of the ST11-KL64 clade in Taiwan and in China merits further investigation by whole-genome sequencing.

Of the 17 isolates, 11 were from outpatients, and six were from hospitalized patients. Of the latter two isolates, T1060144 and T1060393 exhibited similar molecular features (such as capsule type, ST type, carbapenemases, and OMP sequences). The isolates were isolated from different patients in the same ward four months apart. Aside from these two, all the other isolates appeared to be of diverse origins. Some of them may have been transmitted from the intestine of the individuals, as intestinal ESBL-producing Enterobacteriaceae have been suggested to be the source of urinary tract infection [28].

Only three of the 17 isolates produced carbapenemases, suggesting the absence of spreading of carbapenemase genes. Twelve of the isolates contained mutations in one or both of the OMP genes that were examined. Carriage of ESBLs combined with loss-of-function mutations in OMPs appeared to be the primary resistance mechanism in these isolates. The expression levels of efflux pumps and their regulators in the five isolates without any OMP mutations may be classified into two groups. One group showed a slightly increased expression of the efflux pumps examined and exhibited an overexpression of the RamA regulator. The other group showed a lack of overexpression of the efflux genes that were tested. In addition, the inhibition of membrane permeability by PAβN in all the isolates was less severe than that in the reference strain, suggesting the existence of one or more other efflux pumps involved in antibiotic resistance. The involvement of resistance-nodulation-division (RND)-type efflux pumps, such as EefAB [29], KexD [30], and KexEF [31], has been reported.

The overexpression of intrinsic efflux pumps is known to confer antimicrobial resistance [32]. In *K. pneumoniae*, two efflux pumps, AcrAB and OqxAB, have been shown to be associated with resistance to tigecycline [33,34] and nitrofurantoin [35]. Therefore, efflux pump inhibitors (EIs), such as PAβN and quinoxaline, may serve as antibiotic adjuvants to increase the efficacy of antibiotics [36,37,38]. Although many types of EIs have been developed and exhibit potential for application against multidrug-resistant bacteria, their high toxicity to eukaryotic cells limits their clinical use. The discovery and development of better EIs for combinational therapies are still urgently needed.

Plant compounds with antimicrobial activities are candidates for combination therapies with antibiotics to combat multidrug-resistant bacteria [39,40]. For instance, several essential oils extracted from tropical trees in Vietnam have been demonstrated to inhibit both gram-positive and gram-negative bacteria [41,42,43,44]. In particular, essential oils from *Atalantia sessiflora* Guillauminin are effective against *K. pneumoniae* [42], whereas those from *Leoheo domatiophorus* Chaowasku, D.T. Ngo and H.T. Le have been determined to be highly effective against a clinical *Enterococcus faecalis* strain, which is one of the causative agents of complicated UTIs [43]. In addition, essential oils from *Hornstedtia bella* Škorničk showed both antifungal and antibacterial efficacy [41]. These essential oils showed no cell toxicity, and some of them have been utilized in traditional medicine. The potential exists for natural products similar to these ones to be incorporated into therapeutic and/or preventive medicine for urinary tract infection by multidrug-resistant bacteria, such as CRKP.

## Figures and Tables

**Table 1:** Antimicrobial susceptibility tests of 17 carbapenem-resistant *K. pneumoniae* isolates from urine at Taipei Medical University Hospital.

Isolate	Ward ^4^	Age	Isolated Date	CMZ ^1^	CAZ ^1^	CRO ^1^	FEP^1^	IMP ^1^	MEM ^1^	ETP ^1^	GEN ^1^	AMK ^1^	CIP ^1^	LVX ^1^	SXT ^1^	TZP ^1^	COL ^1^	TGC ^2^
T1050022	Ward 7	68	3 December 2016	R (≥32)	R (>16)	I (32)	S (≤2)	R (4)	S (≤1)	R (>4)	R (>8)	S (≤8)	S (≤0.5)	S (≤1)	R (>2/38)	R (>64/4)	WT (1)	S (0.5)
T1060027	ER	82	4 January 2017	R (≥32)	I (8)	R (>32)	R (>16)	I ^3^	S (≤1)	R (>4)	R (>8)	S (≤8)	R (>2)	R (>4)	S ^3^	R (>64/4)	WT (1)	S (1)
T1060063	ER	88	13 February 2017	R (≥32)	R (>16)	I (16)	S (≤2)	R (8)	I (2)	R (>4)	R (>8)	S (≤8)	S (1)	S (≤1)	R (>2/38)	R (>64/4)	WT (1)	S (0.19)
T1060125	Ward 10B	65	21 March 2017	R (≥32)	R (>16)	R (>32)	R (>16)	R (> 8)	R (>8)	R (>4)	R (>8)	R (>32)	R (>2)	R (>4)	S (≤0.5/9.5)	R (>64/4)	WT (1)	S (0.5)
T1060144	Ward 8B	89	4 April 2017	I (32)	R (>16)	R (>32)	R (>16)	R (8)	R (4)	R (4)	R (>8)	S (≤8)	S (1)	S (≤1)	R (>2/38)	R (>64/4)	WT (1)	S (0.75)
T1060283	Ward 8A	95	20 June 2017	R (≥32)	R (>16)	R (>32)	R (>16)	I^3^	S (≤1)	I (1)	R (>8)	R (>32)	R (>2)	R (>4)	R (>2/38)	R (>64/4)	WT (1)	S (0.75)
T1060372	OPD	54	2 August 2017	R (≥32)	R (>16)	S (≤1)	S (≤1)	R (4)	S (≤0.25)	S (0.5)	R (>8)	S (≤8)	S (1)	S (≤1)	R (>2/38)	S (≤4/4)	WT (1)	S (1)
T1060383	ER	88	6 August 2017	R (≥32)	R (>16)	S (≤1)	S (≤1)	I (2)	S (≤0.25)	S (≤0.25)	R (>8)	S (≤8)	R (>2)	R (>4)	S (≤0.5/9.5)	S (≤4/4)	WT (1)	S (0.38)
T1060393	Ward 8B	79	14 August 2017	I (32)	R (>16)	R (>32)	R (>16)	R (> 4)	R (>4)	R (2)	R (>8)	R (>32)	S (1)	S (≤1)	R (>2/38)	R (≥64/4)	WT (≤0.25)	S (0.5)
T1060405 ^5^	ER	81	17 August 2017	R (≥32)	R (>16)	R (16)	S (≤1)	R (4)	S (≤0.25)	I (1)	S (≤2)	S (≤8)	R (>2)	R (>4)	R (>2/38)	I (64/4)	NWT (16)	R (2)
T1060412	OPD	93	15 August 2017	R (≥32)	R (>16)	I (2)	S (2)	R (> 4)	R (4)	R (>4)	S (≤2)	S (16)	R (>2)	R (>4)	S (≤0.5/9.5)	R (≥64/4)	WT (≤0.25)	S (0.25)
T1060431 ^5^	ER	81	26 August 2017	R (≥32)	R (>16)	R (32)	S (2)	R (> 4)	I (2)	R (>4)	S (≤2)	S (≤8)	R (>2)	R (>4)	R (>2/38)	R (≥64/4)	NWT (8)	R (2)
T1060449	Ward 8	55	5 September 2017	R (≥32)	S (4)	S (≤1)	S (≤1)	R (4)	S (≤0.25)	S (0.5)	R (>8)	S (≤8)	S (1)	S (≤1)	R (>2/38)	S (≤4/4)	WT (0.5)	I (1.5)
T1060495	OPD	47	28 September 2017	R (≥ 32)	R (>16)	R (>32)	I (8)	I (2)	S (≤0.25)	S (0.5)	R (>8)	S (≤8)	S (1)	S (≤1)	R (>2/38)	I (64/4)	WT (0.5)	S (0.75)
T1060497	ER	73	25 September 2017	R (≥32)	I (8)	R (>32)	I (8)	I^3^	S (≤0.25)	R (2)	S (≤2)	S (≤8)	R (>2)	R (>4)	S (≤0.5/9.5)	S (≤4/4)	WT (0.5)	S (0.75)
T1060498	OPD	86	20 September 2017	R (≥32)	R (>16)	R (32)	S (≤1)	I ^3^	R ^3^	R (≥2)	R (>8)	S (≤8)	I (2)	S (≤1)	R (>2/38)	I (64/4)	WT (0.5)	S (0.25)
T1060500	OPD	64	22 September 2017	R (≥32)	S (2)	S (≤1)	S (≤1)	R (4)	S (≤0.25)	S (≤0.25)	S (≤2)	S (≤8)	S (1)	S (≤1)	R (>2/38)	S (≤4/4)	WT (0.5)	I (1.5)
			Susceptibility Frequency (%)	0% (0/17)	11.8% (2/17)	23.5% (4/17)	58.8% (10/17)	0% (0/17)	58.8% (10/17)	29.4% (5/17)	29.4% (5/17)	82.4% (14/17)	47.1% (8/17)	52.9% (9/17)	29.4% (5/17)	29.4% (5/17)	88.2% (15/17)	76.5% (13/17)

^1^ Minimal inhibitory concentrations (MICs) of antibiotics except tigecycline were determined by using the microbroth dilution method and interpreted according to Enterobacteriaceae in the CLSI guideline (2018). The MIC values are shown in parentheses. ^2^ The susceptibility of tigecycline was determined by using ETEST^®^ and interpreted based on the EUCAST guideline (2018). The cutoff breakpoint for susceptibility to tigecycline was equal to or less than 1 μg/mL, whereas the MIC value was equal to or more than 2 μg/mL for resistance to tigecycline. ^3^ Interpretation without MIC values was verified by using disk diffusion methods. ^4^ OPD: outpatient department; ER: emergency room. ^5^ These two strains were isolated from the same patient.

**Table 2:** Molecular characterization of 17 carbapenem-resistant *K. pneumoniae* isolates from urine at Taipei Medical University Hospital.

Isolate Name	Sequence Types	Capsule Type	β-Lactamase ^1^					OmpK35 ^2^	OmpK36 ^2^	
			TEM	SHV	AmpC	CTX-M	Carbapenemase	Mutations	Type	Description
T1060027	ST11	KL64	+	+	DHA	Group 9	-	Ser233fsX	C	135 amino acid deletion and frameshift
T1060125	ST11	KL47	+	+	-	Group 9	KPC	Asn29fsX	D	2 amino acid deletion
T1060283	ST11	KL64	+	+	DHA	Group 9	-	Ser233fsX	A	WT
T1060412	ST11	KL64	+	+	DHA	Group 9	-	Ser233fsX	A	WT
T1060497	ST11	KL64	+	+	DHA	Group 9	-	Ser233fsX	A	WT
T1060495	ST219	KL114/125	+	+	DHA	Group 1	-	WT	C	Frameshift
T1060063	ST256	KL47	+	+	DHA	-	-	WT	D	Frameshift
T1060405	ST273	KL15/17/51/52	+	+	DHA	-	-	WT	A	1 missense mutation (Val178Pro)
T1060431	ST273	KL15/17/51/52	-	+	DHA	-	-	WT	A	1 missense mutation (Val178Pro)
T1060372	ST290	KL21	-	+	DHA	-	-	WT	A	7 missense mutations and 1 amino acid insertion
T1060383	ST307	~KL102/149/155 ^3^	-	+	DHA	-	-	Trp79X	D	WT
T1060500	ST412	KL57	-	+	DHA	Group 9	-	WT	C	Frameshift
T1060144	ST461	KL51	-	+	-	Group 9	VIM	WT	D	WT
T1060393	ST461	KL51	+	+	-	Group 9	VIM	WT	D	WT
T1060449	ST469	KL12	+	+	DHA	-	-	WT	A	WT
T1050022	ST1968	KL102/149/155	+	+	DHA	-	-	WT	D	WT
T1060498	~ST4919 /ST5154 ^3^	nd ^4^	+	+	DHA	-	-	WT	C	IS insertion at N-terminal region
Prevalence (Positive/Total)			70.6% (12/17)	100% (17/17)	82.3% (14/17)	52.9% (9/17)	17.6% (3/17)	35.3% (6/17)	A: 7 C: 4 D: 6	A: WT (4); a.a. substitutes (2) and insertion (1) C: frameshift (3); IS insertion (1) D: WT (4); frameshift (1); deletion (1)

^1^ Three carbapenemase genes (*bla*_NDM_, *bla*_IMP_, and *bla*_OXA-48_) and 2 extended-spectrum β-lactamases (*bla*_CTX-M-gp2_ and *bla*_CTX-M-gp8/25_) were not found among these isolates by using polymerase chain reaction. ^2^ The NCBI accession number of the OmpK35 reference was AFR33751.1. The references corresponding to OmpK36 types A to D were QIR56615.1, QHW96390.1, QIV29771.1, and QEI51044.1 in the NCBI database, respectively. Abbreviations: WT, wild type; fsX, frameshift with a premature stop codon (X). ^3^ The symbol “~” indicates proximity to specific types. ^4^ nd: no product after PCR amplification.

## Data Availability

Data is contained within the article or supplementary material.

## Supplementary Materials

The following materials are available online at https://www.mdpi.com/2076-2607/9/2/271/s1, Table S1: Primers used for the determination of multilocus sequence type (MLST) in this study; Table S2: Primers used for the detection of carbapenemases and extended-spectrum β-lactamases in this study; Table S3: Primers used for genes encoding outer membrane proteins and for the detection of gene expression levels in this study.
