# Supplementary material for: Molecular Epidemiology and Characterization of Carbapenem-Resistant Klebsiella pneumoniae Isolated from Urine at a Teaching Hospital in Taiwan
Source: Microorganisms. 2021 Jan 28;9(2):271. doi: 10.3390/microorganisms9020271 (PMC7911515; doi:10.3390/microorganisms9020271)
Supplement: Supplementary file 1 [file microorganisms-09-00271-s001.pdf]

Table S1. Primers used for the determination of multilocus sequence type (MLST) in this study

Gene name	Primer name	Sequences (5′ to 3′ end)	Amplicon size	References
gapA	gapA-F	TGAAATATGACTCCACTCACGG	662	[1]
	gapA-R	CTTCAGAAGCGGCTTTGATGGCTT		
infB	infB-F	CTCGCTGCTGGACTATATTCG	462	
	infB-R	CGCTTTCAGCTCAAGAACTTC		
mdh	mdh-F	CCCAACTCGCTTCAGGTTCAG	756	
	mdh-R	CCGTTTTTCCCCAGCAGCAG		
pgi	pgi-F	GAGAAAAACCTGCCTGTACTGCTGGC	718	
	pgi-R	CGCGCCACGCTTTATAGCGGTTAAT		
phoE	phoE-F	ACCTACCGCAACACCGACTTCTTCGG	602	
	phoE-R	TGATCAGAACTGGTAGGTGAT		
rpoB	rpoB-F	GGCGAAATGGCWGAGAACCA	1075	
	rpoB-R	GAGTCTTCGAAGTTGTAACC		
wzi	wzi_for2	GTGCCGCGAGCGCTTTCTATCTTGGTATTCC	580	[2]
	wzi_rev	GAGAGCCACTGGTTCCAGAAYTTSACCGC		

Table S2. Primers used for the detection of carbapenemases and extended-spectrum β-lactamases in this study

Gene name	Primer name	Sequences (5′ to 3′ end)	Amplicon size (bp)	References
		Primers for carbapenemases		
$\mathit{bla}_{\mathrm{IMP}}$	IMP-F	GGAATAGAGTGGCTTAAYTCTC	233	[3]
	IMP-R	GGTTTAAYAAAACAACCACC		
$\mathit{bla}_{\mathrm{VIM}}$	VIM-F	CCGATGGTGTTTGGTCGC	397	
	VIM-R	CGGTCGAATGCGCAGCAC		
$\mathit{bla}_{\mathrm{OXA\text{-}48}}$	OXA-F	CCCAATAGCTTGATCGCCC	466	
	OXA-R	CATCAAGTTCAACCCAACCG		
$\mathit{bla}_{\mathrm{KPC}}$	KPC-Fm	CGTCTAGTTCTGCTGTCTTGTC	798	
	KPC-Rm	CTTGTCATCCTTGTTAGGCGC		
$\mathit{bla}_{\mathrm{NDM}}$	NDM1-F	ATGGAATTGCCCAATATTATGC	813	[4]
	NDM1-R	TCAGCGCAGCTTGTCGGC		
		Primers for TEM, SHV, CTX-M-group 1, 2, 9, 8/25, CMY, DHA		
$\mathit{bla}_{\mathrm{TEM}}$	MultiTSO-T_for	CATTTCCGTGTCGCCCTTATTC	800	[5]
	MultiTSO-T_rev	CGTTCATCCATAGTTGCCTGAC		
$\mathit{bla}_{\mathrm{SHV}}$	MultiTSO-S_for	AGCCGCTTGAGCAAATTAAAC	713	
	MultiTSO-S_rev	ATCCCGCAGATAAATCACCAC		
$\mathit{bla}_{\mathrm{CTX\text{-}M\text{-}gp1}}$	MultiCTXMGp1_for	TTAGGAARTGTGCCGCTGYA	688	
	MultiCTXMGp1-2_rev	CGATATCGTTGGTGGTRCCAT		
$\mathit{bla}_{\mathrm{CTX\text{-}M\text{-}gp2}}$	MultiCTXMGp2_for	CGTTAACGGCACGATGAC	404	
	MultiCTXMGp1-2_rev	CGATATCGTTGGTGGTRCCAT		
$\mathit{bla}_{\mathrm{CTX\text{-}M\text{-}gp9}}$	MultiCTXMGp9_for	TCAAGCCTGCCGATCTGGT	561	
	MultiCTXMGp9_rev	TGATTCTCGCCGCTGAAG		
$\mathit{bla}_{\mathrm{CTX\text{-}M\text{-}gp8/25}}$	CTX-Mg8/25_for	AACRCRCAGACGCTCTAC	326	
	CTX-Mg8/25_rev	TCGAGCCGGAASGTGTYAT		
$\mathit{bla}_{\mathrm{DHA}}$	MultiCaseDHA_for	TGATGGCACAGCAGGATATTC	997	
	MultiCaseDHA_rev	GCTTTGACTCTTTCGGTATTCG		
Gene name	Primer name	Sequences (5′ to 3′ end)	Amplicon size (bp)	References
$\mathit{bla}_{\mathrm{CMY}}$	MultiCaseCIT_for	CGAAGAGGCAATGACCAGAC	538	[5]
	MultiCaseCIT_rev	ACGGACAGGGTTAGGATAGY		

Table S3. Primers used for genes of outer membrane proteins and for the detection of gene expression levels in this study

Gene name	Primer name	Sequences (5′ to 3′ end)	Amplicon size (bp)	References
		Outer membrane proteins		
ompK35	ompK35for	AACTTATTGACGGCAGTGGC	1,132	[6]
	ompK35rev	TTGGTAAACGATACCCACGG		
ompK36	ompK36for	GCAGTGGCATAATAAAAGGCA	1,144	
	ompK36rev	ACTGGTAAACCAGGCCCA		
		Gene expression levels		
acrB	acrBF	CGATAACCTGATGTACATGTCC	206	[7]
	acrBR	CCGACAACCATCAGGAAGCT		
oqxB	oqxB1806F	GAGCGAGATCGGGATGAATAC	84	[8]
	oqxB1890R	CGGCGTGTTGGTGAACTG		
ramA	ramAF	ATCGTCGAGTGGATTGATGA	85	[9]
	ramAR	AGATGCCATTTCGAATACCC		
rpoB	rpoBF	AAGGCGAATCCAGCTTGTTCAGC	147	[7]
	rpoBR	TGACGTTGCATGTTCGCACCCATCA		

References

1. Diancourt, L.; Passet, V.; Verhoef, J.; Grimont, P.A.; Brisse, S. Multilocus sequence typing of *Klebsiella pneumoniae* nosocomial isolates. *J Clin Microbiol* **2005**, *43*, 4178-4182, doi:10.1128/JCM.43.8.4178-4182.2005.
2. Brisse, S.; Passet, V.; Haugaard, A.B.; Babosan, A.; Kassis-Chikhani, N.; Struve, C.; Decre, D. wzi Gene sequencing, a rapid method for determination of capsular type for *Klebsiella* strains. *J Clin Microbiol* **2013**, *51*, 4073-4078, doi:10.1128/JCM.01924-13.
3. Poirel, L.; Walsh, T.R.; Cuvillier, V.; Nordmann, P. Multiplex PCR for detection of acquired carbapenemase genes. *Diagn Microbiol Infect Dis* **2011**, *70*, 119-123, doi:10.1016/j.diagmicrobio.2010.12.002.
4. Huang, T.W.; Chen, T.L.; Chen, Y.T.; Lauderdale, T.L.; Liao, T.L.; Lee, Y.T.; Chen, C.P.; Liu, Y.M.; Lin, A.C.; Chang, Y.H., et al. Copy Number Change of the NDM-1 sequence in a multidrug-resistant *Klebsiella pneumoniae* clinical isolate. *PloS one* **2013**, *8*, e62774, doi:10.1371/journal.pone.0062774.
5. Dallenne, C.; Da Costa, A.; Decre, D.; Favier, C.; Arlet, G. Development of a set of multiplex PCR assays for the detection of genes encoding important beta-lactamases in Enterobacteriaceae. *The Journal of antimicrobial chemotherapy* **2010**, *65*, 490-495, doi:10.1093/jac/dkp498.
6. Landman, D.; Bratu, S.; Quale, J. Contribution of OmpK36 to carbapenem susceptibility in KPC-producing *Klebsiella pneumoniae*. *Journal of medical microbiology* **2009**, *58*, 1303-1308, doi:10.1099/jmm.0.012575-0.
7. Doumith, M.; Ellington, M.J.; Livermore, D.M.; Woodford, N. Molecular mechanisms disrupting porin expression in ertapenem-resistant *Klebsiella* and *Enterobacter* spp. clinical isolates from the UK. *The Journal of antimicrobial chemotherapy* **2009**, *63*, 659-667, doi:10.1093/jac/dkp029.
8. Bialek-Davenet, S.; Lavigne, J.P.; Guyot, K.; Mayer, N.; Tournebize, R.; Brisse, S.; Leflon-Guibout, V.; Nicolas-Chanoine, M.H. Differential contribution of AcrAB and OqxAB efflux pumps to multidrug resistance and virulence in *Klebsiella pneumoniae*. *The Journal of antimicrobial chemotherapy* **2015**, *70*, 81-88, doi:10.1093/jac/dku340.
9. Veleba, M.; Higgins, P.G.; Gonzalez, G.; Seifert, H.; Schneiders, T. Characterization of RarA, a novel AraC family multidrug resistance regulator in *Klebsiella pneumoniae*. *Antimicrobial agents and chemotherapy* **2012**, *56*, 4450-4458, doi:10.1128/AAC.00456-12.
